# Unsuspected Small-Bowel Crohn's Disease in Elderly Patients Diagnosed by Video Capsule Endoscopy

**DOI:** 10.1155/2018/9416483

**Published:** 2018-01-29

**Authors:** Che-Yung Chao, Carl Frederic Duchatellier, Ernest G. Seidman

**Affiliations:** ^1^Division of Gastroenterology, McGill University Health Center, Montreal, QC, Canada; ^2^Department of Gastroenterology and Hepatology, Princess Alexandra Hospital, Brisbane, QLD, Australia

## Abstract

**Background:**

Video capsule endoscopy (VCE) is increasingly performed among the elderly for obscure bleeding. Our aim was to report on the utility of VCE to uncover unsuspected Crohn's disease (CD) in elderly patients.

**Methods:**

Retrospective review of VCE performed in elderly patients (≥70 y) at a tertiary hospital (2010–2015). All underwent prior negative bidirectional endoscopies. CD diagnosis was based on consistent endoscopic findings, exclusion of other causes, and a Lewis endoscopic score (LS) > 790 (moderate-to-severe inflammation). Those with lower LS (350–790) required histological confirmation. Known IBD cases were excluded.

**Results:**

197 VCE were performed (mean age 78; range 70–93). Main indications were iron deficiency anemia (IDA), occult GI bleeding (OGIB), chronic abdominal pain, or diarrhea. Eight (4.1%) were diagnosed as CD based on the aforementioned criteria. Fecal calprotectin (FCP) was elevated in 7/8 (mean 580 *μ*g/g). Mean LS was 1824. Small-bowel CD detected by VCE led to a change in management in 4/8. One patient had capsule retention secondary to NSAID induced stricture, requiring surgical retrieval.

**Conclusions:**

VCE can be safely performed in the elderly. A proportion of cases may have unsuspected small-bowel CD despite negative endoscopies. FCP was the best screening test. Diagnosis frequently changed management.

## 1. Introduction

Video capsule endoscopy (VCE) is an advanced technology developed to provide noninvasive endoscopic assessment of the small bowel [1]. The capsule consists of an optical camera with light source, batteries, and wireless transmitter. Following ingestion, its movement is propelled by intestinal motility. Luminal images are captured and transmitted to the data recorder worn by the patient. Dedicated computer and software program are used for analysis of the image data.

The main clinical indications for VCE are occult GI bleeding (OGIB), iron deficiency anemia (IDA), suspected or follow-up of Crohn's disease (CD), suspected small-bowel tumor, surveillance of polyposis syndromes, refractory celiac disease, and protein losing enteropathy [2]. VCE is usually used in conjunction with standard esophagogastroduodenoscopy and colonoscopy. It may also be used in preference to other imaging modalities including computer tomography or magnetic resonance enterography or push or balloon assisted enteroscopy, depending on the clinical scenario [3, 4].

VCE is increasingly performed in the elderly population, particularly for IDA and OGIB, due to its superior diagnostic accuracy compared to cross-sectional imaging as well as its noninvasive nature when compared to enteroscopy in a population with significant comorbidities [5].

CD is a chronic relapsing inflammatory bowel disease involving any segment of the GI tract. The age of onset is ordinarily between 15 and 40 years. However, some studies suggested a bimodal peak at a later age [6]. It was recently reported that, compared with patients with adult-onset CD, patients with elderly onset CD were more likely to have isolated colonic disease and a nonstricturing, nonpenetrating phenotype but less likely to have perianal complications [7]. There is however very limited data on newly diagnosed CD in elderly patients uncovered by VCE, thus prompting this retrospective study evaluating the relevant clinical parameters and outcomes in this distinctive population.

## 2. Patients and Methods

A retrospective review was performed on all patients who underwent VCE at the McGill University Health Centre between January 2010 and September 2015. All patients aged 70 or above without prior diagnosis of IBD were included in the study. All indications for VCE were acceptable other than for follow-up of CD. Furthermore, all included patients needed to have had prior and recent (within 1 year) negative bidirectional GI endoscopies.

The diagnosis of small-bowel CD was based on consistent endoscopic findings and exclusion of other potential causes of small-bowel inflammation such as infections, celiac disease, or drugs such as nonsteroidal anti-inflammatory drugs (NSAID) or telmisartan in the previous month. In addition, patients were included if the Lewis endoscopic severity score (LS, Table 1) [8] was >790 (indicating moderate-to-severe inflammation). For cases with a lower LS (350–790), histological confirmation of CD was necessary. Capsule retention was defined as delayed capsule excretion for more than 2 weeks, or requiring retrieval.

Patient demographics, clinical information, diagnostic results, and management outcomes were retrieved through their electronic medical records for analysis. Serum C-reactive protein (CRP) and fecal calprotectin (FCP) results prior to VCE were also collected, if available.

All VCE were performed with PillCam SB2/SB3 (Given Imaging Ltd., Yokneam, Israel) following standard 24-hour clear fluid diet and 2-liter polyethylene glycol based bowel preparation. Images were interpreted with the use of RAPID viewer (Given Imaging Ltd., Yokneam, Israel). LS was calculated with the integrated calculator in the RAPID viewer in accordance with previously validated criteria [8].

## 3. Results

**Figure 1:**
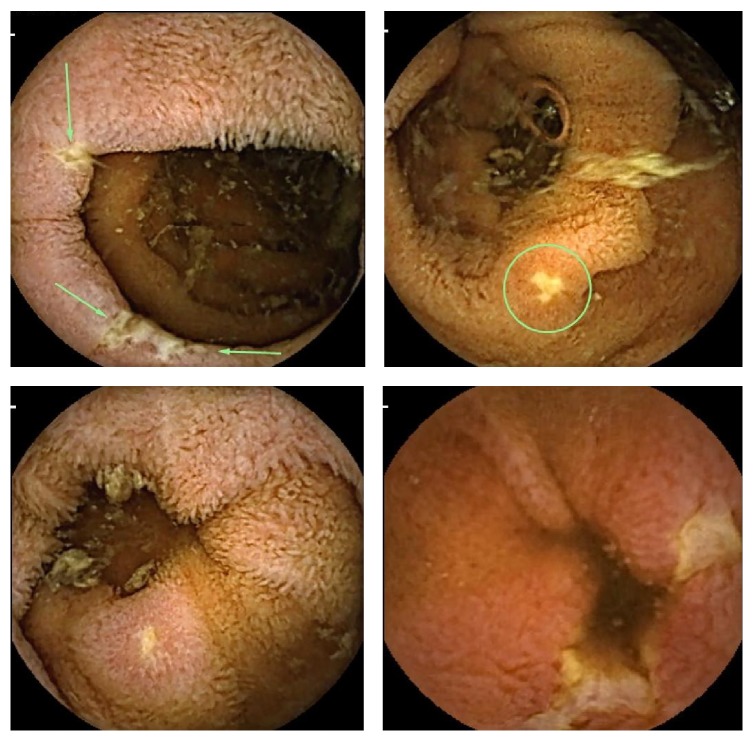
Representative video capsule endoscopy images of small-bowel Crohn's disease.

A total of 197 VCE were performed for elderly patients aged 70 or above during this period. Eight patients (4.1%) were diagnosed with CD using the aforementioned criteria. Representative images are shown in Figure 1. The mean age in this elderly small-bowel CD (SBCD) group is 75 (range 70–87) with 6 females and 2 males. Three patients had IDA either alone, with chronic abdominal symptoms (pain and/or diarrhea), or with a history of ankylosing spondylitis (AS). Two patients had chronic abdominal pain and diarrhea. One patient was being investigated for overt OGIB. The remaining two patients were asymptomatic and had been referred to exclude SBCD in light of their clinical history of AS.

The mean FCP was 580 *μ*g/g (range 15–818), with all but one patient having a value ≥600. Mean CRP was 3.9 mg/l (range 0.96–5.8) and mean white blood cell count (WBC) was 6.3 × 10^9^ /L (range 5.1–8.6). The mean combined LS was 1824 (range 768–6680) with the majority of the patients having moderate-to-severe inflammation and one with strictures. One patient with LS < 790 had subsequent histological confirmation of CD following further endoscopic investigation with balloon assisted enteroscopy. CRP (Pearson correlation *r* = 0.71, *p* = 0.29), WBC (*r* = 0.56, *p* = 0.92), and FCP (*r* = 0.38, *p* = 0.53) were not statistically correlated to the LS. Cross-sectional imaging results prior to VCE were available in 3 of the 8 CD patients, all of which were standard computed tomography of the abdomen without a specific enterography protocol. Two of these patients had abdominal symptoms. No definitive findings of CD were found in all of the scans.

One patient had an incomplete study due to delayed capsule passage related to an inflammatory SB stenosis. The capsule was eventually passed within a few days, without the need for retrieval. No other complications were observed.

The VCE results led to therapeutic changes in 4 out of 8 SBCD patients. Ensuing treatments included methotrexate, adalimumab, budesonide, and 5-amino salicylic acid. A patient was continued on mycophenolate mofetil and cyclosporine for a previous renal transplant. One other patient was managed expectantly for her mild asymptomatic CD. Follow-up was not available for the remaining 2 patients (Table 2).

The non-CD group consists of 189 elderly patients with a mean age of 77 (range 70–93) and 51.9% were female. The majority of the patients (94.7%) were referred for investigation of IDA, OGIB, or a combination thereof (Table 3). 41.8% had a normal study. Otherwise, the most common findings were angiodysplasia and minor nonspecific inflammatory changes (erythema, villous changes, aphthous ulcers, and edema) (Table 4).

One patient in the non-CD group had capsule retention due to a NSAID induced inflammatory stricture which required surgical retrieval. This patient did not undertake patency capsule testing priorly as there were no significant symptoms or risk factors suggestive of intestinal obstruction. Five patients had an incomplete study, two resulting from delayed gastric emptying and 3 from delayed intestinal transit without an obvious cause.

## 4. Discussion

This single center retrospective review showed that, despite negative bidirectional GI endoscopies, a small proportion of elderly patients may have unsuspected SBCD uncovered by VCE. Previous population studies have demonstrated that although only approximately 5% of CD cases are diagnosed in patients aged 60 and over, the incidence is increasing around the world [9, 10]. Accurate diagnosis based on VCE in this population remains challenging in light of other confounding factors such as NSAID use as well as the restricted capacity for invasive tissue confirmation. Nevertheless, this study highlights an important finding on VCE in the elderly population which is likely to rise in view of the widespread use of this technology in the aging population.

There has been a growing focus on issues relating to elderly IBD patients. However, often studies fail to distinguish patients with older-onset CD versus others who transition into older age following diagnosis at an earlier age, even though the prior group may exhibit different clinical phenotypes and natural history compared to their younger counterparts [7, 11]. A recent meta-analysis demonstrated that older-onset CD patients are more likely to have colonic disease (OR 2.56, 95% CI 1.88–3.48), inflammatory phenotype (OR 1.19, 95% CI 1.07–1.33), and a similar likelihood of stricturing disease (OR 0.90, 95% CI 0.67–1.20), but lower risk for penetrating phenotype (OR 0.48, 95% CI 0.33–0.69) and less perianal disease (0.64, 95% CI 0.56–0.80) [12]. A recent multicenter retrospective cohort study of patients with IBD also found that, compared with younger patients, those with elderly onset CD were more likely to have isolated colonic involvement, a nonstricturing, and nonpenetrating phenotype and less likely to have perianal complications or to receive immunosuppressants [7]. Rates of bowel resection, and both colonic and extracolonic malignancies, did not differ based on the age of IBD onset. It is notable that Ananthakrishnan et al. also observed that despite the lower rate of immunomodulator use (OR 0.44, 95% CI 0.33–0.57), presumably due to age related concerns, the rate of surgery (OR 0.70, 95% CI 0.40–1.22) was similar compared to younger patients [12]. This suggests that the disease pattern in the elderly is no less benign than in their younger counterparts and that timely diagnosis along with appropriate treatment may prevent complications and need for surgery in the elderly population with more comorbidities.

The majority of patients in this study had moderate-to-severe inflammation on VCE and most required a change in management. Optimal therapeutic approach in the elderly CD population continues to be a contentious issue and is often based on expert opinion, with limited robust data. The main issues reside with the concerns relating to the risks of infection and malignancy along with other adverse effects in this population where existing risks associated with the medications are further compounded by advanced age [13–15]. In addition, elderly patients are generally excluded from clinical trials, rendering management expertise in this age group to be of lower quality. It is thus not uncommon to find a preferential use of medications such as budesonide and 5-amino salicylic acid in this population for their perceived lower side effect profile. No clear difference in efficacy nonetheless has been demonstrated with the use of thiopurine or methotrexate in the elderly [16]. However, the CESAME study showed that incidence rate of lymphoproliferative disorders in IBD patients aged over 65 with persisting thiopurine exposure was significantly increased [13].

The response and tolerance to antitumor necrosis factor alpha therapy have been reported to vary with age. Some studies have suggested similar long term response compared to younger patients whereas others have shown a lower response rate along with higher chance of discontinuation among elderly onset disease [17, 18]. Additionally, therapeutic options are also hindered by polypharmacy, drug interactions, and other pertinent issues in this cohort, such as heart failure [19]. Anti-TNF alpha therapy is contraindicated in patients with classes III-IV New York Heart Association heart failure [20]. These factors should be taken into consideration when forming a tailored and individualised approach to the management of elderly CD patients. Gut selective anti-integrin therapy such as vedolizumab may theoretically provide a superior safety profile in this higher risk population [21].

In this study, the majority of CD cases had a significantly elevated FCP, suggesting that this may serve as a helpful predictive biomarker for the presence of small-bowel inflammation on VCE in the elderly. Other serum biomarkers including CRP and WBC performed poorly. FCP is a well-established diagnostic test in detecting the presence of GI tract inflammation and is widely used as a screening test [22]. It is yet to be conclusively demonstrated whether there is a difference in its diagnostic accuracy when assessing for small bowel versus colonic CD, as previous studies have shown conflicting results [23, 24]. A recent meta-analysis involving 7 clinical studies with 463 patients examined the accuracy of FCP in detecting SBCD on VCE. At the cut-off level of 50 *μ*g/g, the sensitivity, specificity, and diagnostic odds ratio were 0.89, 0.55, and 10.3, respectively, in patients suspected of SBCD. This suggests that the probability of SBCD below this cut-off is low and thus may be used as a screening tool to forego VCE use in those with negative bidirectional GI endoscopies in the right clinical context. Two additional studies published since also provided evidence in support of this notion [25, 26]. On the other hand, some studies have shown that the correlation between FCP and objective assessment of the SB inflammation such as the validated LS is equivocal or moderate at best [27, 28]. This may have been influenced by the retrospective nature of these studies or inherently within the LS itself, as the presence of a stricture without an associated inflammation will significantly increase the score but may not be associated with a corresponding increase in FCP. There are nevertheless no studies to date examining the use of FCP in the elderly population as a screening test prior to VCE. Variation in FCP with age was reported in healthy individuals [29]. Children aged 2–9 y had significantly higher concentrations than subjects aged ≥10 y. Adults ≥ 60 years had a higher concentration than those aged 10–59 y. Further prospective studies would be helpful in determining whether stratification with FCP may lead to improved outcomes and cost-effectiveness in the elderly.

Finally, this study demonstrates that the feasibility and safety of VCE in the elderly population are comparable to younger onset CD [30]. Only one non-CD patient with NSAID enteropathy experienced capsule retention, requiring intervention. The patency capsule was developed to assess intestinal patency prior to administration of VCE in an attempt to minimize capsule retention [31]. It is highly predictive of successful VCE completion, comparable to other cross-sectional imaging [32]. However, the decision to use a patency capsule varies between institutions. None of the patients in this study received patency testing as they did not exhibit any high-risk features predictive of retention. Currently, it is recommended to use a patency capsule in those with clinical or radiological features of SB stenosis, such as history of intestinal obstruction, surgery, or radiation, as well as those with established CD [33]. A recent study has challenged whether all CD patients need patency testing since the nonselective use of patency capsule in established CD patients did not improve retention rates in comparison with restricted patency testing in high-risk patients only [34].

The Korean Gut Image Study Group has developed guidelines for use of VCE in the following situations: diagnosis of OGIB, small-bowel preparation for VCE, diagnosis of CD, and diagnosis of small-bowel malignancy [35]. They concluded that VCE is the most sensitive diagnostic modality for detecting mucosal lesions in patients with suspected or established CD and that VCE is useful for diagnosing CD after negative colonoscopy and small-bowel radiology when there is a strong suspicion of CD. They recommended small-bowel radiology or the patency capsule test before VCE in patients with suspected or established CD. The European evidence-based consensus for endoscopy in IBD also recommends cross-sectional imaging or patency capsule testing before VCE in patients with established CD [36].

The main limitations of the study are those inherent within a retrospective study of which missing data, including potential confounding factors, and the relatively small SBCD sample size impact the ability to comprehensively characterize this patient cohort. In addition, most of the CD diagnosis was not confirmed by tissue sampling in light of the accessibility issues within the elderly population therefore potentially being exposed to misclassification bias. Nonetheless, this should be minimized by the inclusion criteria, including consistent VCE appearance of CD, exclusion of other differential diagnoses and moderate-to-severe inflammation, or tissue confirmation for those with only mild inflammation on VCE. Furthermore, VCE interpretation in this study was performed by an expert reader, thus reducing potential interobserver variations. The Lewis score was routinely used prospectively in assessing degree of small-bowel inflammation for all VCE performed at the McGill University Health Centre, which ensured all eligible cases were captured. Finally, although regular NSAID use was an exclusion criterion, 1 of 8 elderly patients with SBCD on VCE was on low dose ASA (80 mg/d).

## 5. Conclusions

Video capsule endoscopy can be safely performed in the elderly population where a proportion of these patients may have unsuspected SBCD despite negative conventional bidirectional GI endoscopies, resulting in change in management. Further studies in this unique group would assist with identifying predictive clinical and biochemical parameters for risk stratification.

## Figures and Tables

**Table 1:** Lewis video capsule endoscopy scoring index.

Parameters	Number	Longitudinal extent	Descriptor
*Villous appearance* (separate score for each small bowel tertile)	Normal: 0 Edematous: 1	Short segment: 8 Long segment: 12 Whole tertile: 20	Single:1 Patchy:14 Diffuse:17
*Ulcer * (separate score for each small bowel tertile)	None: 0 Single: 3 Few: 5 Multiple: 10	Short segment: 5 Long segment: 10 Whole tertile: 15	<1/4—9 1/4–1/2—12 >1/2—18
*Stenosis * (for whole study only)	None: 0 Single: 14 Multiple: 20	Ulcerated: 24 Nonulcerated: 2	Traversed:7 Not traversed: 10
*Total score*: maximum tertile score {[(villous parameter × extent × descriptor) + (ulcer parameter × extent × size)] for tertile 1 or [(villous parameter × extent × descriptor) + (ulcer parameter × extent × size)] for tertile 2 or [(villous parameter × extent × descriptor) + (ulcer parameter × extent × size)] for tertile 3} + (stenosis number × ulcerated × traversed).			

Reprinted with permission from Gralnek et al., Alimentary Pharmacology & Therapeutics, 2008 [8].

**Table 2:** Baseline demographics, indication for VCE, clinical parameters, and outcomes for elderly Crohn's cohort.

Elderly SBCD	8 patients
Age: mean ± SD/range (years)	72 ± 6 (70–87)
Gender	6 females : 2 males
IDA	1/8
OGIB	1/8
Abdominal symptoms	2/8
AS	2/8
IDA + abdominal symptoms	1/8
IDA + AS	1/8
Mean fecal calprotectin ± SD/range (*µ*g/g)	580 ± 326 (15–818)
Mean CRP ± SD/range (mg/l)	3.9 ± 2.1 (0.96–5.8)
Mean Lewis score ± SD/range	1824 ± 1986 (768–6680)
Capsule retention	0
Other complications	1 (incomplete study)
Change in management	4/6 (5-amino salicylate acid/methotrexate/ adalimumab/budesonide)
Lost to follow-up	2/8

SBCD: small-bowel Crohn's disease; SD: standard deviation; *N*: number; IDA: iron deficiency anemia; OGIB: occult gastrointestinal bleeding; AS: ankylosing spondylitis.

**Table 3:** Indication for video capsule endoscopy in elderly patients.

Non-CD patients	189
Age: mean ± SD/range (years)	77 ± 5 (70–93)
Gender	91 females : 98 males
IDA	128 (67.7%)
Overt OGIB	32 (16.9%)
Abdominal symptoms	7 (3.7%)
AS	2 (1.1%)
IDA + abdominal symptoms	1 (0.5%)
IDA + overt OGIB	19 (10.1%)

CD: Crohn's disease; *N*: number; SD: standard deviation; IDA: iron deficiency anemia; OGIB: occult gastrointestinal bleeding; AS: ankylosing spondylitis.

**Table 4:** Diagnostic findings of video capsule endoscopy in elderly patients (excluding Crohn's disease cohort).

Non-CD patients	189
Normal	79 (41.8%)
Angiodysplasia	63 (33.3%)
Celiac disease	4 (2.1%)
Mass lesions	6 (3.2%)
Nonspecific changes	31 (16.4%)
Angiodysplasia/Polyp	5 (2.6%)
Angiodysplasia/Celiac	1 (0.5%)
Capsule retention	1 (0.5%)
Incomplete study	5 (2.6%) (2 due to gastroparesis)
Other complications	0

CD: Crohn's disease.
